# A Case of Best Disease Accompanied by Pachychoroid Neovasculopathy

**DOI:** 10.4274/tjo.galenos.2019.38073

**Published:** 2019-09-03

**Authors:** Figen Batıoğlu, Özge Yanık, Sibel Demirel, Çağatay Çağlar, Emin Özmert

**Affiliations:** 1 Ankara University Faculty of Medicine, Department of Ophthalmology, Ankara, Turkey; 2 Hitit University Faculty of Medicine, Department of Ophthalmology, Çorum, Turkey

**Keywords:** Best disease, optical coherence tomography angiography, pachychoroid neovasculopathy

## Abstract

The aim of this case presentation is to describe ocular findings of a 22-year-old patient with Best vitelliform macular dystrophy accompanied by pachychoroid neovasculopathy. Color fundus photography, fundus autofluorescence (FAF), optical coherence tomography (OCT), and optical coherence tomography angiography (OCTA) images were reviewed. Fundoscopic examination showed bilateral yellowish vitelliform-like submacular deposits. FAF revealed these deposits as hyperautofluorescent spots. OCT showed flat irregular pigment epithelial detachments corresponding to these submacular deposits. OCT showed choroidal thickening and dilatation of the large outer oval choroidal vessels. Fundus fluorescein angiography could not be performed because the patient was pregnant. En face OCTA images of the choriocapillaris illustrated the choroidal neovascular network. In this case report, we describe for the first time the coexistence of Best vitelliform macular dystrophy and pachychoroid neovasculopathy with OCTA images enabling visualization of the neovascular network in a patient with contraindication for fluorescein angiography.

## Introduction

Best vitelliform macular dystrophy (Best disease) is characterized by the presence of a vitelliform macular lesion leading to the classic egg-yolk appearance in genetically predisposed individuals.^1^ The *BEST1* gene (formerly known as *VMD2*), which encodes the protein bestrophin-1 and is located on the 11q12-q13, is responsible for this disease.^2^ Bestrophin is a transmembrane protein found on the basolateral side of retinal pigment epithelium (RPE) cells^3^ and is responsible for calcium-dependent chloride transport.^4^

A severe complication of a Best vitelliform lesion is the development of secondary choroidal neovascularization (CNV), the exact mechanism of which is unknown. Especially when there is a sudden decrease in visual acuity, the development of neovascular tissue should be suspected. Conventional dye angiography should be performed to visualize the CNV. However, dye injection may cause anaphylactic and urticarial reactions in patients both with and without a history of allergy, and hepatic diseases, hemodialysis, and pregnancy are strong relative contraindications for dye injection.^5^ In the presence of such a contraindication, the best alternative is optical coherence tomography angiography (OCTA), which enables noninvasive visualization of the chorioretinal microvasculature layer by layer.

There are few studies in the literature reporting the microvasculature details of a Best lesion with OCTA.^6,7^ To the best of our knowledge, this is the first report describing the coexistence of a Best lesion with bilateral pachychoroid neovasculopathy.

## Case Report

**Figure 1:**
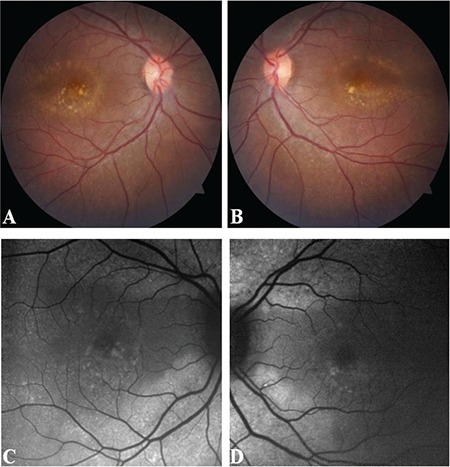
Multimodal imaging findings of the patient: (A, B) Color fundus photographs showing bilateral yellowish submacular vitelliform deposits and tiny yellowish spots located all around the fundus. (C, D) Fundus autofluorescence imaging revealed hyperautofluorescent spots corresponding to the yellowish material in the color photographs

A 22-year-old female was referred to our retina clinic due to the detection of submacular yellowish deposits during routine ophthalmological examination. Other than being 14 weeks pregnant, she had no systemic medical conditions or history of ocular disease. She complained that her vision had worsened in the last few months. Best corrected visual acuity was 20/63 in the right eye and 20/25 in the left eye. Anterior segment examination was unremarkable. Posterior segment examination showed bilateral yellowish vitelliform-like submacular deposits and tiny yellowish spots throughout the fundus (Figure 1). Fundus autofluorescence (FAF) imaging (Heidelberg Retina Angiograph 2^®^; Heidelberg Engineering Inc., Heidelberg, Germany) revealed these deposits as hyperautofluorescent foci. Optical coherence tomography (OCT) revealed flat irregular pigment epithelial detachments (PED). Choroidal thickening and dilation of the large outer oval choroidal vessels were also detected.

**Figure 2:**
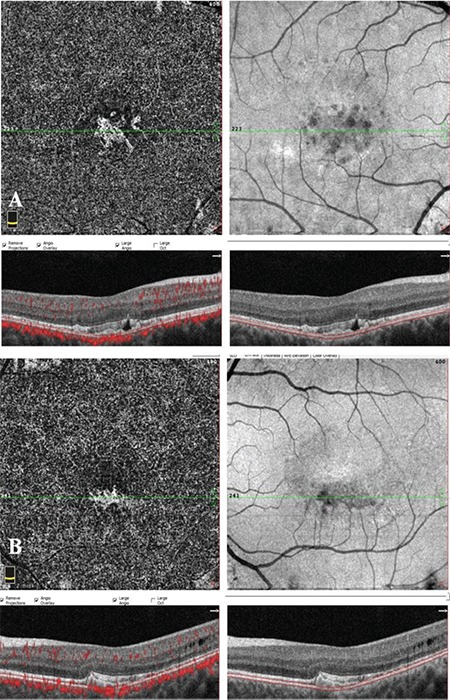
Optical coherence tomography angiography images showing the choriocapillary layer and corresponding B-scans with red flow overlay and segmentation boundary lines. Optical coherence tomography angiography images successfully illustrated the choroidal neovascular network in both the (A) right and (B) left eye. Optical coherence tomography revealed flat, irregular pigment epithelial detachments

Fluorescein and indocyanine green angiography could not been performed because the patient was pregnant. Therefore, the retinal and choroidal vasculature was evaluated with OCTA using the RTVue-XR Avanti OCTA System (Optovue Inc, Fremont, CA). Choriocapillaris en face OCTA images illustrated a dense, highly branching choroidal neovascular network with polypoidal dilations in both eyes (Figure 2). Upon family examination, RPE changes, vitelliform deposits, and tiny peripheral yellowish flecks were detected on the retina of her brother. No treatment could be applied due to her pregnancy, so the patient was scheduled for follow-up visits. However, she did not attend follow-up visits.

## Discussion

Best vitelliform macular dystrophy typically presents in the first two decades of life. However, atypical presentations of Best disease with late development of vitelliform lesions have also been reported.^8,9^ Hormonal, hemodynamic, vascular, and immunological changes occur during pregnancy that can be associated with ocular changes or worsening of preexisting conditions.^10^ Our patient experienced her first visual complaints in the first trimester of pregnancy. She did not report having any visual symptoms during adolescence.

Although the responsible gene and protein have been identified, the exact mechanism underlying Best disease is unknown. The responsible protein, bestrophin-1, is believed to serve as a calcium-dependent chloride channel, bicarbonate transporter, and volume regulator in the plasma membrane, and as a chloride channel or calcium sensor in the endoplasmic reticulum.^11,12,13,14^ A molecular study reported disrupted fluid flux and increased accrual of autofluorescent material after long-term photoreceptor outer segment feeding, delayed rhodopsin degradation, and altered calcium responses in Best disease.^15^ The authors indicated that calcium signaling and oxidative stress are critical contributors to the clinical manifestation of Best disease. RPE dysfunction leads to impaired turnover of the shed photoreceptor outer segments, leading to an accumulation of this material in the subretinal space. FAF hyperautofluorecense corresponding to the yellowish material seen in fundus examination and flat irregular PEDs on OCT supported the association of this material with the photoreceptor outer segments. Due to accumulated material and subretinal fluid formation, the loss of the direct apposition between the RPE and photoreceptor outer segments may result in further accumulation of subretinal vitelliform material.

The superiority of the OCTA to fluorescein angiography in the visualization of the CNV in Best disease was reported previously.^6^ It was also indicated that fluorescein angiography might grossly underestimate the actual neovascular area present.^6^ The masking effect of the vitelliform lesion may limit the visualization of the underlying neovascular tissue. In our case, CNV is seen clearly within the vitelliform material in OCTA scans. Another advantage of OCTA is that it does not require dye injection, which eliminates all nephrotoxicity, anaphylaxis reactions, and contraindication due to pregnancy, as in our case. However, its inability to detect leakage limits its use in determining the activity of CNV lesions. Recent studies have tried to determine OCTA pattern characteristics in order to differentiate active versus quiescent CNV.^16,17^

Best disease was previously associated with choroidal thinning.^18,19^ However, our patient had pachychoroid spectrum disease characteristics with increased choroidal thickness.^20^ Flat irregular PEDs were present on OCT images. The high incidence (74%) of type 1 CNV in pachychoroid spectrum diseases with flat irregular PEDs was reported in another of our studies previously.^21^ Consistent with these data, OCTA showed a dense, highly branching choroidal neovascular network in both eyes of the patient. To the best of our knowledge, this is the first report describing the association of Best disease with pachychoroid neovasculopathy. Further studies are needed in order to determine whether the coexistence of these two diseases is a coincidence or a consequence of a common mechanism in their pathophysiology.
